# Genome-Wide Analysis of Genetic Diversity and Selection Signatures in Zaobei Beef Cattle

**DOI:** 10.3390/ani14162447

**Published:** 2024-08-22

**Authors:** Liangyu Shi, Pu Zhang, Qing Liu, Chenhui Liu, Lei Cheng, Bo Yu, Hongbo Chen

**Affiliations:** 1 Laboratory of Genetic Breeding, Reproduction and Precision Livestock Farming & Hubei Provincial Center of Technology Innovation for Domestic Animal Breeding, School of Animal Science and Nutritional Engineering, Wuhan Polytechnic University, Wuhan 430023, China; liangyu_shi@whpu.edu.cn (L.S.); z15515092327@163.com (P.Z.); liu3996406@163.com (Q.L.); 2 Institute of Animal Science and Veterinary Medicine, Wuhan Academy of Agricultural Sciences, Wuhan 430208, China; lchhcl890621@sina.com (C.L.); chenglei@wuhanagri.com (L.C.)

**Keywords:** Zaobei cattle, genomic diversity, population structure, selection signatures

## Abstract

**Simple Summary:**

Positive natural selection increases the frequency of beneficial genetic variations, promoting an organism’s adaptability and survival in specific environments. In this study, we conducted whole-genome resequencing and population genetic structure analysis to investigate the genetic diversity of Zaobei cattle, local yellow cattle from China. By comparing Zaobei cattle with Simmental cattle, we identified several genomic regions exhibiting positive selection. Gene annotation of these regions revealed key traits of Zaobei cattle, such as heat tolerance, fertility, and superior meat quality. These findings not only help understand the characteristics of Zaobei cattle but also provide valuable genetic information for future breeding programs, thereby improving their production efficiency and economic value.

**Abstract:**

This investigation provides a comprehensive analysis of genomic diversity and selection signatures in Zaobei beef cattle, an indigenous breed known for its adaptation to hot and humid climates and superior meat quality. Whole-genome resequencing was conducted on 23 Zaobei cattle, compared with 46 Simmental cattle to highlight genetic distinctions. Population structure analysis confirmed the genetic uniqueness of Zaobei cattle. Using methods such as DASDC v1.01, XPEHH, and θπ ratio, we identified 230, 232, and 221 genes through DASDC, including hard sweeps, soft sweeps, and linkage sweeps, respectively. Coincidentally, 109 genes were identified when using XPEHH and θπ ratio methods. Together, these analyses revealed eight positive selection genes (*ARHGAP15*, *ZNF618*, *USH2A*, *PDZRN4*, *SPATA6*, *ROR2*, *KCNIP3*, and *VWA3B*), which are linked to critical traits such as heat stress adaptation, fertility, and meat quality. Moreover, functional enrichment analyses showed pathways related to autophagy, immune response, energy metabolism, and muscle development. The comprehensive genomic insights gained from this study provide valuable knowledge for breeding programs aimed at enhancing the beneficial traits in Zaobei cattle.

## 1. Introduction

**Figure 1:**
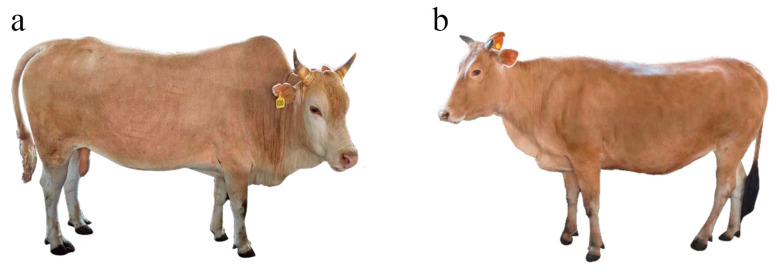
Photographs of Zaobei cattle: (**a**) bull; (**b**) cow.

Cattle are one of the most important livestock species in the world. By providing milk and dairy products, labor, meat, leather, and other products, they have become indispensable to human society. Carcass quality, meat quality, and reproduction performance are widely recognized as critical economic traits influencing the production of beef cattle. Zaobei cattle (Figure 1), a local yellow cattle breed distributed in Hubei Province in China, are known for their muscular build, superior meat quality, and exceptional reproductive performance. These cattle are predominantly found in rolling hills, plains, and undulating terrain, with elevations ranging from 70 to 780 m above sea level and the highest temperature above 40 degrees Celsius in summer. The breeding area falls within a subtropical continental monsoon climate zone, influenced annually by the southeast monsoon, resulting in a humid and rainy climate. The geographical and climatic features of the region play a significant role in shaping the adaptability and phenotypic traits of Zaobei cattle. Unlike Zaobei cattle, Simmental cattle originated from middle Europe [1]. In terms of herd management, the traditional practice primarily involves extensive free-range grazing. This approach allows the cattle to forage naturally, which aligns with the local agricultural practices and contributes to their distinctive meat quality and overall health. Therefore, these cattle exhibit great heat tolerance and can adapt to hot and humid environments in Southern China. The meat of Zaobei cattle is prized for its flavor, with the back and hind leg areas exhibiting distinctive marbling due to fat deposition, where small and thin veins and plexus fatty layers resemble marble, known as “snowflake beef”, a typical representative of high-end raw meat. With the rising demand for high-quality beef recently, the economic value of Zaobei cattle is apparently increasing. Due to their heat tolerance and high-quality beef production, it is of great importance to conserve and utilize Zaobei cattle genetic resources to enhance their productivity and economic and social benefits.

Whole-genome sequence data provide the possibility for a precise analysis of complex traits across many breeds. Selection has significantly changed the phenotypic characteristics of livestock, enhancing their environmental adaptability, along with the yield and quality of animal products [2,3,4]. This process, through positive selection, has promoted the accumulation of beneficial mutations in the genome, leaving distinctive imprints called selection signatures [5]. Identifying traits influenced by selection has become a powerful tool for detecting selection signature [6], aiding in revealing the biological processes behind biological evolution [7,8], productivity [9,10,11], and functional characteristics [12,13,14]. Researchers have developed methods to study these selected traits, each varying in the indicators and statistical inference methods [15,16,17,18,19]. However, they are generally based on evaluating the differences in allele or haplotype frequencies between populations. By identifying hard sweeps and soft sweeps signals, we can assess the genomic regions and their associated genes controlling quantitative traits, thereby gaining an understanding of the biological mechanisms behind these traits

Although Zaobei cattle’s genetic diversity has been studied, it has mainly focused on the introgression and demographic history [20]. Existing research suggests that Chinese yellow cattle breeds have high genetic diversity, with genotypic differences existing among different breeds [21,22]. However, the knowledge on the genetic constitution for Zaobei cattle is still limited, particularly in research of genetic variations related to adaptability and economic traits. The present study enhances the comprehension of genetic mechanisms underlying economically significant traits in cattle through the application of whole-genome resequencing. Our aim is to characterize the genetic diversity of Zaobei cattle by assessing their diversity, adaptability, and traits associated with production efficiency. This knowledge provides valuable insights for efficient implementation of cattle breeding programs and contributes to designing more effective hybridization or making improvement plans for breeding.

## 2. Materials and Methods

### 2.1. Sample Collection and Sequencing

To ensure that samples represent the genetic diversity of the entire population, we specifically selected 18 Zaobei cattle for our study, focusing on different families and geographical distributions within their native region. Genomic DNA was extracted from ear tissues. Two paired-end libraries were constructed and sequenced using the Huada MGI-T7 platform, with an average coverage depth of roughly ~20× per individual. For comparison purposes of the selection sweeps and genetic diversity, we used 5 Zaobei cattle and 46 Simmental cattle obtained from the NCBI Sequence Read Archive repository under the BioProject accession numbers PRJNA379859, PRJNA238491, and PRJNA343262.

### 2.2. Whole-Genome Sequencing Data Alignment and Variant Calling

The raw data from the two cattle breeds were initially processed using Trimomatic v0.39 [23] to remove adapter sequences and low-quality bases. Reads with an average quality below 15 in a 5-base window or leading/trailing bases below a score of 5 were discarded, retaining sequences exceeding 50 bases after trimming. Subsequent to cleaning, reads were aligned to the reference cattle genome ARS-UCD1.3 (GCA_002263795.3) using the BWA-MEM (version 0.7.17) [24] with default parameters. Samtools [25] was used to sort and index aligned sequences, and duplicate reads were identified and removed with the command MarkDuplicates from Picard v2.27.1 [26] “https://broadinstitute.github.io/picard/ (1 May 2022)” to reduce redundancy and potential biases in subsequent analyses.

SNP identification was performed using GATK v4.2.6.1 [27]. Initially, individual BAM files, processed to remove duplicates and recalibrate base quality, were analyzed with “HaplotypeCaller” to generate GVCF files per sample. These GVCFs were merged into multi-sample VCF files using “CombineGVCFs”. Joint genotyping across all samples was then conducted with “GenotypeGVCFs”, followed by SNP selection using “SelectVariants”. SNP filtering criteria were applied using the “VariantFiltration” tool to minimize false positives. The employed filtration parameters included quality by depth (<2.0), mapping quality (<40), strand odds ratio (>3.0), and Fisher strand score (>60), phred-scaled quality scores (QUAL) below 30.0, as well as those demonstrating marked deviations in mapping quality rank sum tests (<−12.5) or read position rank sum tests (<−8.0). SNP imputation and phasing were performed using BEAGLE v5.4 [28,29], utilizing default parameters. Genetic variations were annotated using ANNOVAR v4.7 [30]. SNPs with a minor allele frequency (MAF) of greater than 0.05 were considered in this analysis.

### 2.3. Population Structure and Genetic Analysis

The distance matrix, derived from SNP data using PLINK v1.90 [31], was used to construct a phylogenetic tree with the neighbor-joining method. The Interactive Tree of Life (iTOL v6) [32] was employed to facilitate visualization. ADMIXTURE v1.3.0 software [33] was used to conduct an admixture analysis, utilized for the investigation of population substructure. Genetic clusters (K) were explored from 2 to 3. To further scrutinize the genome-wide linkage disequilibrium (LD) within each breed, we computed the average *r*^2^ values for pairwise markers with PopLDdecay [34] with default settings.

### 2.4. Detection of Selection Signatures

Previous studies have shown that artificial intelligence methods possess greater power in detecting selection signatures compared to traditional statistical approaches [35]. To identify the signatures of selection driven by artificial selection and genetic adaptation to the local environment, we employed Domain Adaptation Sweep Detection and Classification (DASDC v1.01) [36] for intra-population analyses to detect genomic selection signals in Zaobei cattle. Unlike other AI methods, DASDC explicitly considers the issue of simulation data quality, reducing its impact on model performance, and has proven effective in livestock populations where genetic parameters are difficult to infer accurately. Compared to traditional methods, it can identify more complex patterns and signals, including soft sweeps. Additionally, this model is capable of distinguishing between hard and soft sweeps, providing more nuanced evidence for understanding the evolutionary processes in organisms. Following previous research [37,38], we simulated five classes of 100k genomic fragments (population size of 100), including hard selective sweeps, soft selective sweeps, hard linkage sweeps, soft linkage sweeps, and neutral, with 3000 cases for each class (total 15,000 cases). The dataset was divided into training, validation, and test sets in an 8:1:1 ratio. The model was trained using the training set and real genomic data, evaluated on the test set, and subsequently used for detecting and classifying genomic selection signatures in the Zaobei cattle genome.

To further delineate the positive selection signatures influenced by domestication or environmental adaptation in Zaobei cattle, we employed additional inter-population genetic differentiation methods. We performed XPEHH [39] using selscan v2.0.2 software [40,41]. All these calculations were assessed in 50 kb windows with 20 kb steps to identify significant genomic regions. Additionally, the average nucleotide diversity (π) and population genetic differentiation (F_ST_) [42] were assessed using high-quality autosomal SNPs. These SNPs were examined within non-overlapping 50 kb windows with a step size of 20 kb across all bovine autosomes using VCFtools version v0.1.16 [43]. Moreover, the θπ ratios were calculated.

### 2.5. Functional Annotation of Selection Signatures and Enrichment Analysis

Significant genomic regions identified by the top 1% of results from DASDC and θπ ratio, and XPEHH > 2.58 [44], were annotated using the Ensembl BioMart tool [45]. Regions, defined by the overlap of signals from at least two methodologies, located candidate genes mapped to the ARS-UCD1.2 cattle reference genome [46]. To elucidate the biological implications of these genes, functional annotation was performed using the WebGestaltR R package [47,48], which facilitated Gene Ontology (GO) [49] and Kyoto Encyclopedia of Genes and Genomes (KEGG) pathway enrichment analyses [50]. A *p*-value threshold of 0.05 was used for these analyses, with the results visualized using the ggplot2 package [51] in R v4.2.3.

## 3. Results

### 3.1. Whole-Genome Resequence and SNP Identification

We conducted whole-genome sequencing (WGS) on the Zaobei, a geographically diverse breed in China, and the Simmental cattle breed. High-throughput sequencing generated 1 Tb. The data yielded from the 46 Simmental cows have an average sequencing depth of 7.73×. The analyses focused on biallelic SNPs identified, and the annotation result was shown in Supplementary Materials Table S1. After quality control, including the filtering based on minor allele frequency and call rate, 25,085,581 SNPs were identified and retained for further analysis. The sequencing reads were aligned to the reference genome, achieving an average alignment rate above 94%.

### 3.2. Population Structure and Linkage Disequilibrium Analysis

**Figure 2:**
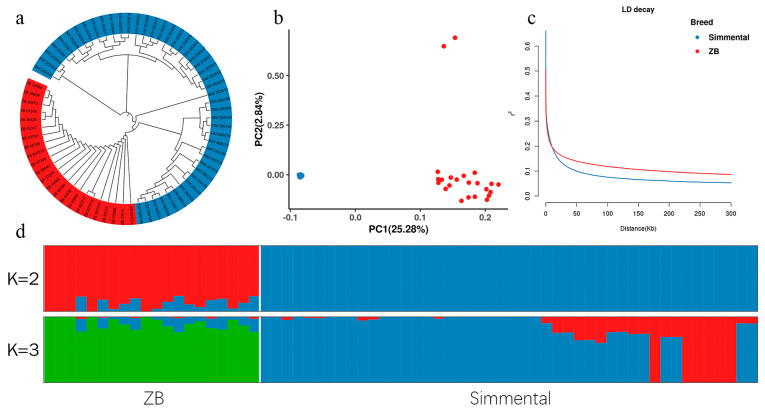
Population structure analysis: (**a**) neighbor-joining tree of the relationships in these populations; (**b**) principal component analysis; (**c**) decay of *r*^2^ with pair-wise SNP marker distances in Zaobei (ZB) and Simmental cattle; (**d**) ancestry component analysis of these cattle breeds using ADMIXTURE v1.3.0 with K = 2 and K = 3.

To elucidate the genetic structure and diversity within the population of Zaobei and Simmental cattle through comprehensive analyses, including principal component analysis (PCA), ADMIXTURE v1.3.0, π, and linkage disequilibrium (LD). PCA was performed to capture the main axes of genetic variation among the sampled individuals. The first two principal components (PC1 and PC2) together explained 28.12% of the total genetic variance, indicating distinct genetic separations between Zaobei and Simmental cattle (Figure 2b). PC1, in particular, accounted for 25.28% of the variance, effectively delineating the two breeds. neighbor-joining (NJ) and maximum likelihood (ML) trees were constructed to visualize the phylogenetic relationships among the sampled individuals (Figure 2a,d). These phylogenetic trees corroborated the PCA results, showing clear clusters corresponding to each breed, supporting the genetic distinctiveness of the populations studied.

LD analysis showed different patterns between two breeds. Zaobei cattle exhibited higher LD values compared to Simmental cattle, suggesting higher linkage within their genomes (Figure 2c). The decay of LD with physical distance was quantified, indicating a sharper decline in LD values for Simmental cattle at 0.7 kb compared to 3.1 kb for Zaobei cattle.

**Figure 3:**
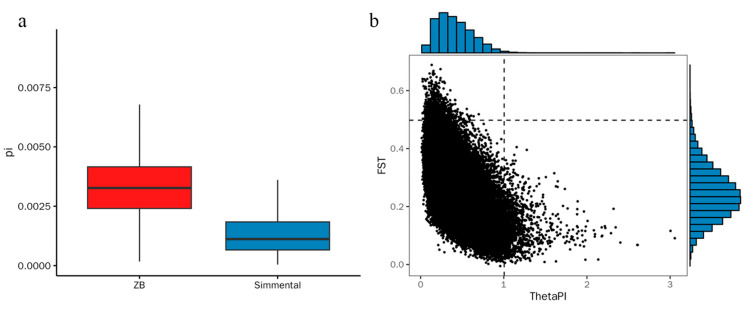
Nucleotide diversity and differentiation in Zaobei (ZB) (red) and Simmental Cattle (blue): (**a**) genome-wide distribution of nucleotide diversity for each breed; (**b**) identification of putatively selected genomic regions in cattle populations using both fixation index (F_ST_) and π ratio methods.

The assessment of genetic diversity and differentiation was carried out by calculating the nucleotide diversity (π) and pairwise fixation index (F_ST_) values (Figure 3). Zaobei cattle exhibited higher π values, indicative of their extensive genetic base, compared to Simmental cattle. The average F_ST_ value is 0.247363, emphasizing the moderate to high genetic differentiation between two breeds.

### 3.3. Signature of Detection in the Zaobei Cattle and Gene Annotationn

**Figure 4:**
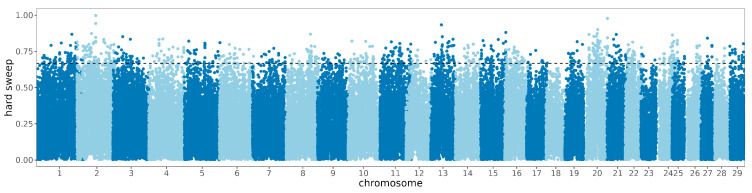
Manhattan diagram of the selective characteristics of hard sweep signatures.

We then utilized the Domain Adaptation Sweep Detection and Classification (DASDC) model for discerning selection signals within the genome of Zaobei cattle. The DASDC model effectively identified regions exhibiting hard sweeps, soft sweeps, and linkage sweeps. In detecting hard sweeps, 857 significant regions and 230 genes with strong selection signals were found, primarily on chromosomes 2 and 20 (Figure 4). The highest selection signal on chromosome 20 was annotated with the *SPEF2* gene. The *SPEF2* gene is a well-known gene in cattle because of its association with important traits, such as adaptation to heat stress [52], fertility [53,54], temperament [55], as well as milk production and composition [56]. This gene, *SPEF2*, was annotated in GO:0048702, GO:0048854, and GO:0069541, which are related to the embryonic neurocranium, brain morphogenesis, and respiratory system development. On chromosome 2, a significant selection signal was linked to the *CMKLR2* gene, also known as *GPR1*, found to be expressed in the bovine ovary, thereby affecting fertilization. Genes annotated to significant regions were found to be largely enriched in autophagy (bta04140), human immunodeficiency virus 1 infection (bta05170), mitophagy, Fc gamma R-mediated phagocytosis (bta04666), and other related immune pathways (Supplementary Figures S1 and S2).

**Figure 5:**
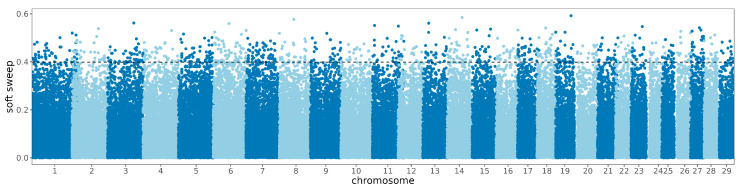
Manhattan diagram of the selective characteristics of soft sweep signatures.

Regarding soft sweeps, 857 remarkable regions were identified, encompassing a total of 232 genes (Figure 5). A functional enrichment analysis concerning these genes situated in the selected regions of the Zaobei cattle genome was conducted. Subsequent Gene Ontology (GO) and Kyoto Encyclopedia of Genes and Genomes (KEGG) enrichment analyses illuminated various pathways associated with fertilization, adaptability in severe environments, fat accumulation, and meat quality, such as the AMPK signaling pathway (bta04152), genitalia development (GO:0048806), and potassium ion transmembrane transport (GO:0071805) (Supplementary Figures S3 and S4).

**Figure 6:**
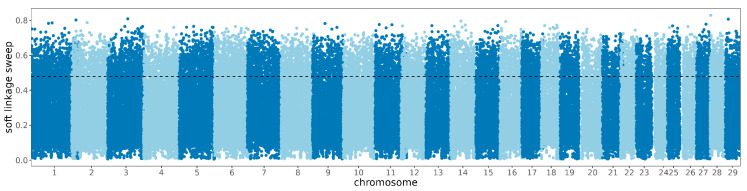
Manhattan diagram of the selective characteristics of soft linkage signatures.

In identifying soft linkage sweeps, regions showed significant selection signals due to linkage with directly selected regions. A total of 856 significant regions were identified, annotated with 221 genes (Figure 6). These genes include *PIGK*, *MUC20*, *ATP6V1A*, *KCNU1*, and *GSE1*, which are associated with fat deposition [57], fertility [58,59,60,61,62] and temperament [63]. The genes annotated within the significant regions of soft linkage sweeps link to the calcium signaling pathway (bta04020), primary immunodeficiency (bta05340), and T cell receptor signaling pathway (bta04660), possibly correlating with differentiation and metabolism of fat cells and reproduction (Supplementary Figures S5 and S6).

### 3.4. Selective Sweep and Enrichment Analysis between Breeds

In order to explore the selection signatures of the two breeds, selective sweep analyses were performed on each strain using a consistent identification method to detect the selected regions of Zaobei and Simmental.

**Figure 7:**
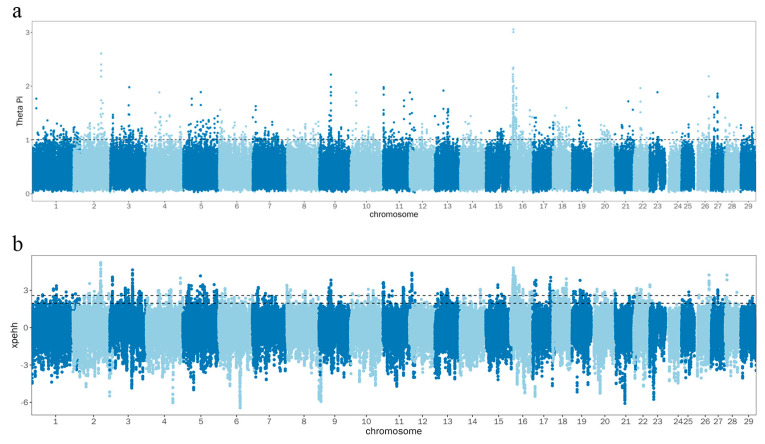
Select sweep analysis: (**a**) significant loci from θπ; (**b**) significant loci from XPEHH.

The θπ method detected 1197 significant regions and annotated them to 474 genes, while the XPEHH method uncovered 632 significant regions and annotated them to 201 genes. Of these significant regions, 280 exist in multiple XPEHH and θπ genomic windows, predominantly on chromosome 16 (Figure 7). In these overlapping regions, the two methods together recognized 109 genes, identifying them as prospective candidate genes for Zaobei cattle. Gene Ontology (GO) and Kyoto Encyclopedia of Genes and Genomes (KEGG) analyses revealed that these genes enrich pathways associated with the regulation of the secretion of proteases, peptides, cytokines, and other secretions, energy metabolism, immunity, and selenium-related pathways, for example, positive regulation of protein secretion (GO:0050714), glycolysis process (GO:0006096), ATP generation from ADP (GO:0006757), and HIF-1 signaling pathway (bta04066) (Supplementary Figures S7 and S8). Notably, the *ABI2* gene was annotated at the point of the maximum signal value, while the *GPAT2* gene was annotated in a significant region on chromosome 11. The *ABI2* gene is regulated during embryonic development, and its aberrant splicing isoforms may adversely affect early embryonic development and subsequent reproductive traits. This could include issues such as reduced embryo implantation rates, developmental delays, or fetal abnormalities [64]. Additionally, *ABI2* is associated with muscle development [65].

## 4. Discussion

The characterization of genetic diversity and population structure is requisite to reveal the adaptive and productive ability of cattle populations, thereby having significant implications for future efforts in genetic enhancement and conservation. The principal component (PC) and admixture analyses differentiate Zaobei cattle from European taurine breeds. The Simmental breed is associated with the Eurasian taurine ancestral component, while the Zaobei breed is associated with Chinese indicine ancestral component [20]. Phylogenetic tree and population genetic analysis in this study further support the point that Zaobei and Simmental are different categories, revealing their clear genetic difference. This not only reflects their respective geographic origins and genetic history, but also may indicate their genetic variations under different environmental factors.

Under natural and artificial selection, cattle have formed various breeds and populations with different phenotypic characteristics and have adapted to a variety of local environments, including both hot and cold conditions. Positive selection is a form of natural selection that enhances the adaptability and survival ability of organisms in specific environments by increasing the frequency of beneficial genetic variations, helping us understand how specific genetic variations affect the adaptive traits of organisms [66]. This selection mechanism can be divided into several categories, mainly including hard sweeps and soft sweeps. Hard sweeps refer to the rapid spread and fixation of newly emerged beneficial mutations within populations, which quickly fix advantageous traits in livestock, such as disease resistance or increased productivity. Unlike hard sweeps, soft sweeps refer to the existing genetic variations that have become beneficial due to environmental changes and usually increase in frequency, but do not achieve fixation. The presence of soft sweeps suggests a more diverse genetic response to selective pressures, which can be beneficial for the long-term adaptability and resilience of livestock populations. This mode of selection allows for rapid adaptation and preserving genetic diversity. Zaobei cattle have excellent reproductive performance and high meat quality and can adapt well to hot and humid environments. In this study, we implemented three distinct methodologies: DASDC, which includes hard sweep, soft sweep, soft linkage sweep, θπ, and XPEHH. The purpose of using different methodologies was to identify selection patterns in Zaobei cattle and pinpoint genetic variations and potential genes linked with reproduction, meat quality, and thermal tolerance. Nonetheless, no candidate regions were identified simultaneously by various selection-type methods. This might be due to the statistical differences between the methods that allow them to reveal different types of selection signatures across different timescales [67]. To reduce the false-positive rate, studies suggest that positive selection regions in the genome should be identified by at least two statistical methods [68]. Therefore, we defined the regions shortlisted by at least two methods.

By utilizing three methods (hard sweep, θπ, and XPEHH), we detected *ARHGAP15* and *USH2A* genes in the cattle genome. *ARHGAP15* has been found to be related to physiological traits associated with tropical adaptability in multiple breeds of cattle [69]. As one of the regulatory proteins of the Rho GTPase family, *ARHGAP15* is a Rho GTPase activating protein that participates in various biological processes such as dynamic changes in the cytoskeleton, cell polarity, movement, and proliferation. Therefore, it may play a key role in the tropical adaptability of cattle by influencing these cellular processes [70]. The *USH2A* gene affects cattle hair color, which may be linked with adaptive traits or survival advantages [71]. In addition, the *ZNF618* and *PDZRN4* genes were detected in both hard sweep and θπ. *ZNF618* is a zinc finger protein; its expression in bovine leukocytes is downregulated due to Theileria annulata infection, which may involve the host’s response to the pathogen [72]. In the northwest, northeast, and north parts of China, this gene is associated with the prevalence of theileriosis; while Zaobei cattle are mostly spread in the central China region, positive selection analysis shows a significant signal for the *ZNF618* gene, indicating its potential role in adaptive evolution. The *PDZRN4* gene is highly expressed in human abdominal adipose tissue [73]. Moreover, this gene is considered an important functional candidate gene for intramuscular fat content in pigs [74,75].

The *SPATA6* and *ROR2* genes were detected by soft sweep and θπ. *SPATA6*, a gene noted for its evolutionary conservation and specific expression in the testis, encodes a protein integral to the formation of segmented columns and the capitulum. These structures play an essential role in the sperm connecting piece, related to the developing flagellum to the sperm head during the phase of spermiogenesis. Disruption of *SPATA6* in mice induces the production of acephalic spermatozoa, resulting in male sterility [76]. Additionally, *SPATA6* is an important functional molecule of spermatogenesis, regulating the proliferation, apoptosis, and testosterone biosynthesis of Hu sheep Leydig cells [77].

The genes *KCNIP3* and *VWA3B* were identified using three methods: soft linkage sweep, θπ, and XPEHH. The *KCNIP3* gene may be involved in melatonin-regulated inflammatory hyperalgesia and reduced morphine tolerance [78]. The *KCNIP3* protein exhibits similarities with melatonin at multiple levels, including oscillatory responses to circadian rhythms in the pineal gland and retina, as well as interactions with key enzymes regulating melatonin synthesis. It have been shown that disruptions in maternal circadian rhythms influence fetal development and reproductive health through impacts on the maternal circadian clock and related melatonin rhythms [79]. *VWA3B* with milk glycosylated kappa-casein percentage and higher differentiation among breeds for deletions [80,81].

We recognize that our study does not include functional validation data, which limits our ability to conclusively link the identified selection signatures with specific phenotypic traits in Zaobei cattle. Future research will aim to incorporate detailed phenotypic data and functional validation to better understand the biological implications of these genetic markers.

## 5. Conclusions

This research reveals significant positive selection in Zaobei beef cattle, distinguishing them from Simmental cattle and enhancing their adaptation to hot and humid environments. Key genomic regions under positive selection were identified, namely *ARHGAP15*, *ZNF618*, *USH2A*, *PDZRN4*, *SPATA6*, *ROR2*, *KCNIP3*, and *VWA3B*. These genes are associated with essential traits like heat stress adaptation, fertility, and meat quality, indicating a shaped genetic landscape of Zaobei cattle under selective pressures. Functional enrichment analyses further highlighted pathways related to autophagy, immune response, energy metabolism, and muscle development, highlighting the biological significance of the identified selection signatures. These insights support breeding strategies to make full use of valuable traits of Zaobei cattle for environmental adaptability and high-quality beef production.

## Data Availability

The data presented in this study are available on request from the corresponding author. The data are not publicly available to preserve the privacy of the data.

## Supplementary Materials

The following supporting information can be downloaded at https://www.mdpi.com/article/10.3390/ani14162447/s1: Figure S1: GO enrichment analysis of candidate genes from hard sweeps of DASDC. The red, green, and blue parts represent biological process, cellular component, and molecular function, respectively; Figure S2: Enrichment of the top 30 KEGG pathways in candidate genes derived from hard sweeps in DASDC; Figure S3: GO enrichment analysis of candidate genes from soft sweeps of DASDC. The red, green, and blue parts represent biological process, cellular component, and molecular function, respectively; Figure S4: Enrichment of the top 30 KEGG pathways in candidate genes derived from soft sweeps in DASDC; Figure S5: GO enrichment analysis of candidate genes from soft linkage sweeps of DASDC. The red, green, and blue parts represent biological process, cellular component, and molecular function, respectively; Figure S6: Enrichment of the top 30 KEGG pathways in candidate genes derived from soft linkage sweeps in DASDC; Figure S7: GO enrichment analysis of candidate genes from XPEHH and θπ. The red, green, and blue parts represent biological process, cellular component, and molecular function, respectively; Figure S8: Enrichment of the top 30 KEGG pathways in candidate genes derived from XPEHH and θπ. Table S1: Distribution of the identified biallelic SNPs in 69 cattle genomes within various genomic regions.
