# Supplementary material for: Genome-Wide Analysis of Genetic Diversity and Selection Signatures in Zaobei Beef Cattle
Source: Animals (Basel). 2024 Aug 22;14(16):2447. doi: 10.3390/ani14162447 (PMC11350888; doi:10.3390/ani14162447)
Supplement: Supplementary file 1 [file animals-14-02447-s001.zip › animals-3141638-supplementary.pdf]

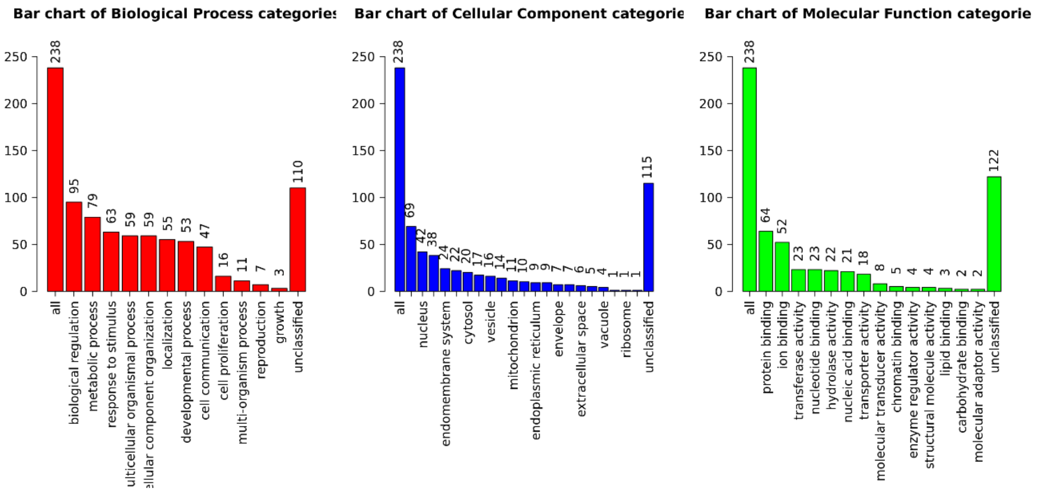

Figure S1. GO enrichment analysis of candidate genes from hard sweeps of DASDC. The red, green, and blue parts represent biological process, cellular component, and molecular function, respectively.

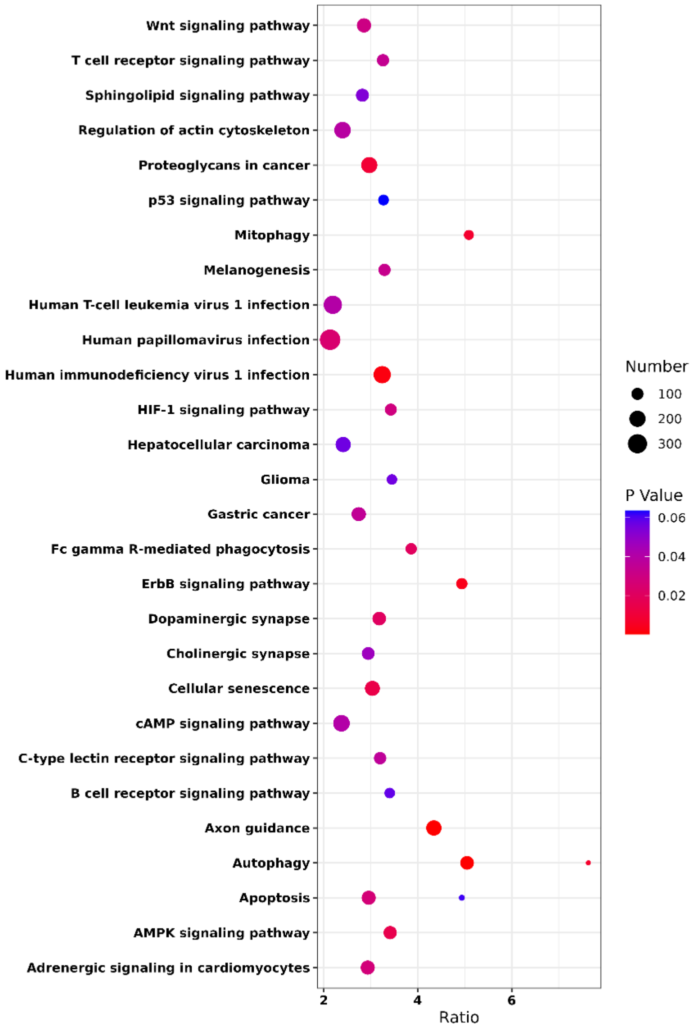

Figure S2. Enrichment of the top 30 KEGG pathways in candidate genes derived from hard sweeps in DASDC.

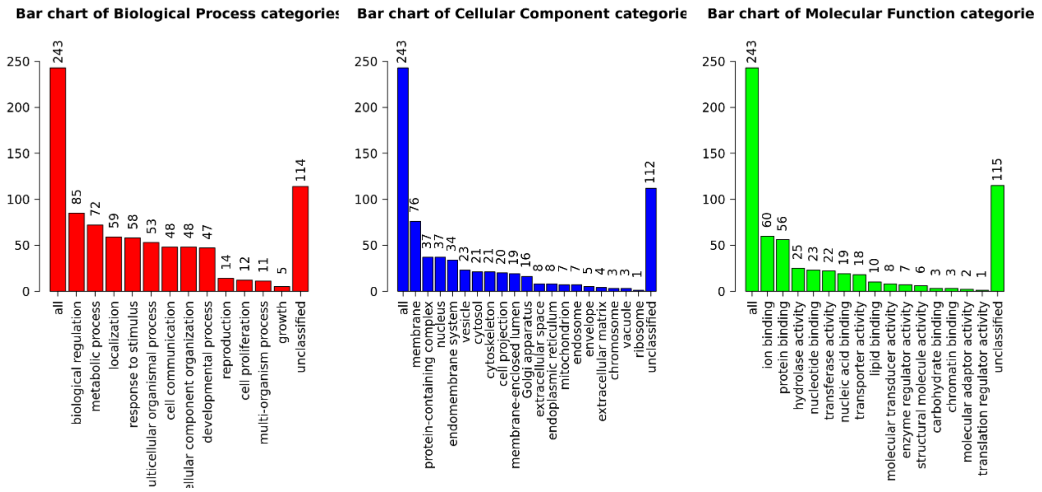

Figure S3. GO enrichment analysis of candidate genes from soft sweeps of DASDC. The red, green, and blue parts represent biological process, cellular component, and molecular function, respectively.

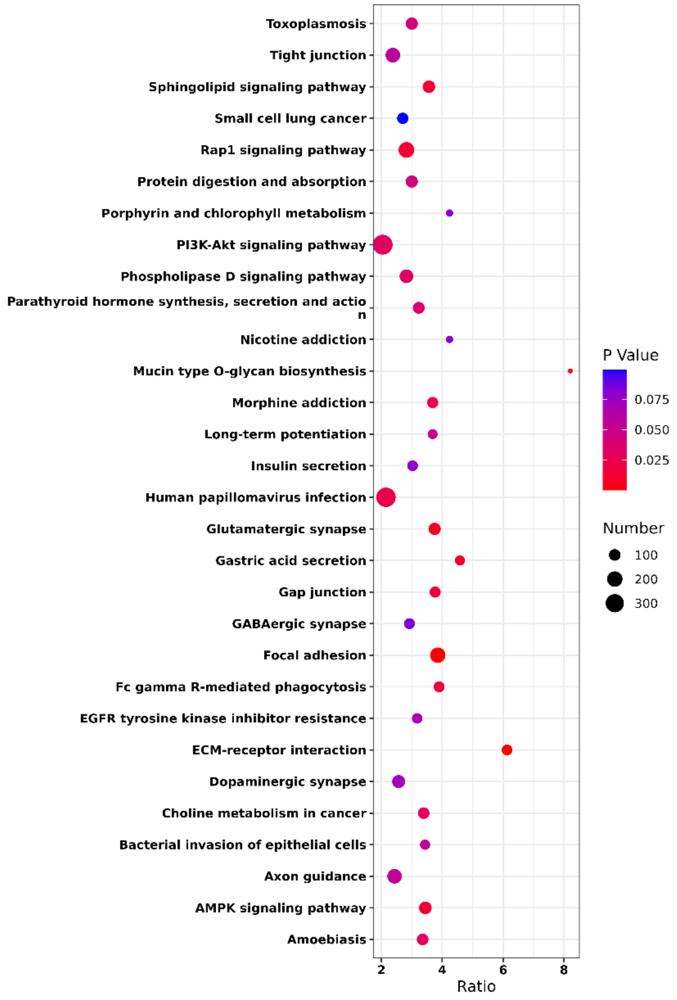

Figure S4. Enrichment of the top 30 KEGG pathways in candidate genes derived from soft sweeps in DASDC.

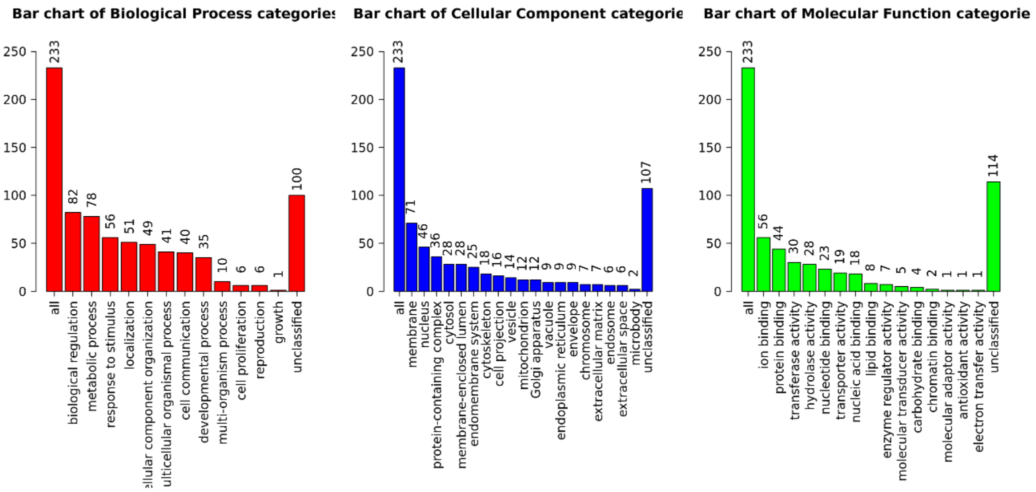

Figure S5. GO enrichment analysis of candidate genes from soft linkage sweeps of DASDC. The red, green, and blue parts represent biological process, cellular component, and molecular function, respectively.

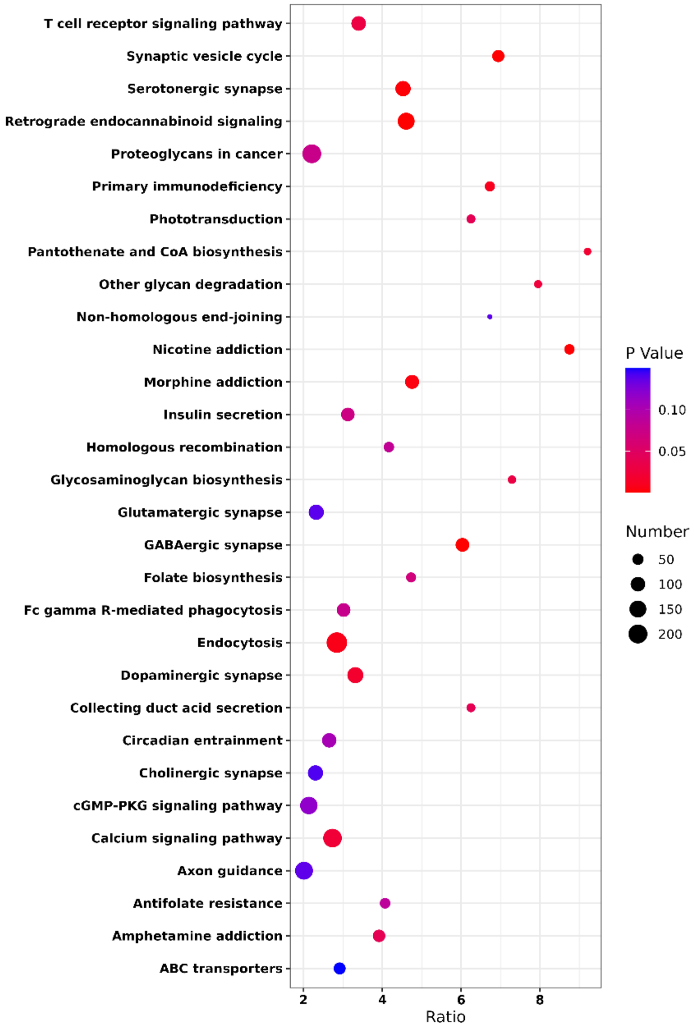

Figure S6. Enrichment of the top 30 KEGG pathways in candidate genes derived from soft linkage sweeps in DASDC.

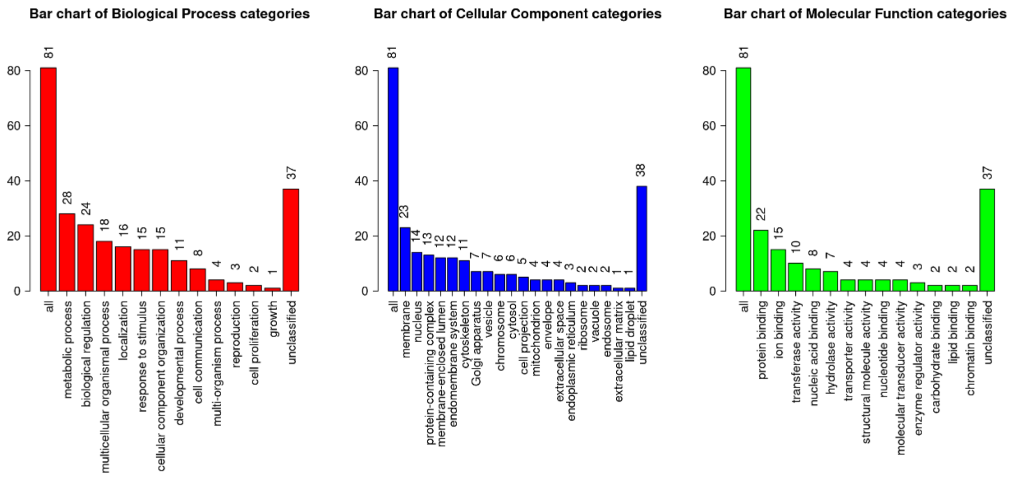

Figure S7. GO enrichment analysis of candidate genes from XPEHH and θπ. The red, green, and blue parts represent biological process, cellular component, and molecular function, respectively.

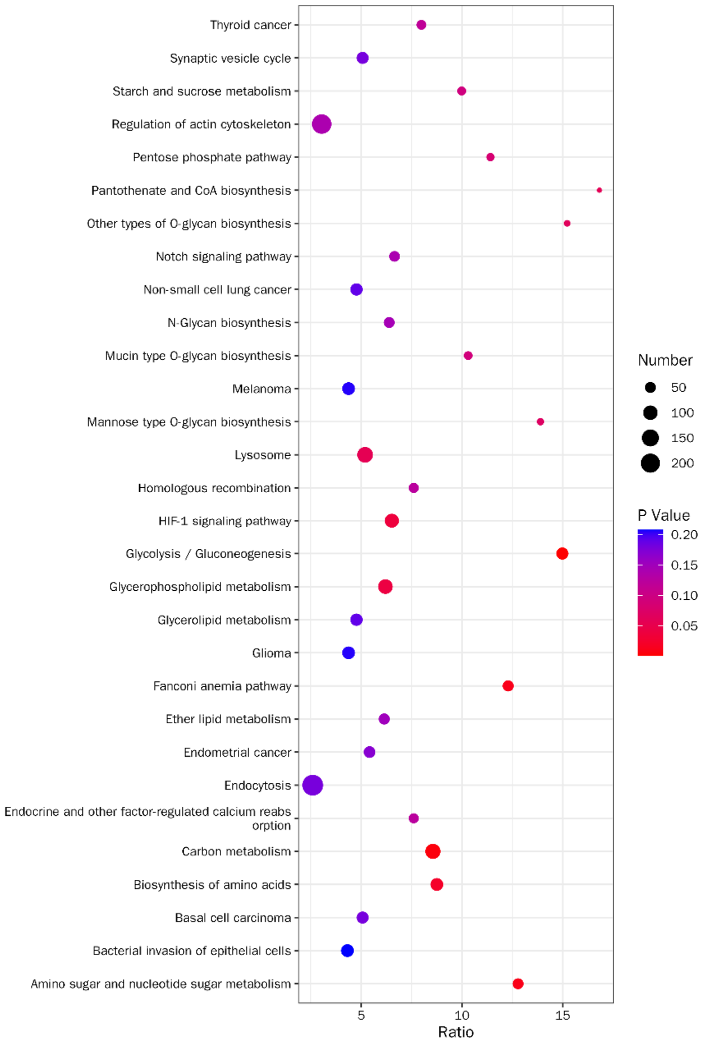

Figure S8. Enrichment of the top 30 KEGG pathways in candidate genes derived from XPEHH and θπ.

Table S1. Distribution of the identified biallelic SNPs in 69 cattle genomes within various genomic regions

Genomic regions	Number of biallelic SNPs	Proportion of biallelic SNPs
downstream	761747	0.86%
exonic	1031560	1.17%
exonic;splicing	553	0.00%
intergenic	45306927	51.21%
intronic	35655521	40.30%
ncRNA_exonic	543339	0.61%
ncRNA_exonic;splicing	604	0.00%
ncRNA_intronic	3504931	3.96%
ncRNA_splicing	2351	0.00%
splicing	10049	0.01%
upstream	707263	0.80%
upstream;downstream	46906	0.05%
UTR3	606325	0.69%
UTR5	281021	0.32%
UTR5;UTR3	9152	0.01%
total	88468249	100.00%

Note: The proportion of SNPs in different categories.
